# Decline in Japanese Encephalitis, Kushinagar District, Uttar Pradesh, India

**DOI:** 10.3201/eid2008.131403

**Published:** 2014-08

**Authors:** Prashant Ranjan, Milind Gore, Sriram Selvaraju, K.P. Kushwaha, D.K. Srivastava, Manoj Murhekar

**Affiliations:** Indian Council of Medical Research, National Institute of Epidemiology, Chennai, India (P. Ranjan, S. Selvaraju, M. Murhekar);; Indian Council of Medical Research, National Institute of Epidemiology, Gorakhpur, India (M. Gore);; Baba Raghav Das Medical Collage, Gorakhpur (K.P. Kushwaha, D.K. Srivastava)

**Keywords:** acute encephalitis syndrome, Japanese encephalitis, Kushinagar India, viruses, vector-borne infections

**To the Editor:** Kakkar et al. recently concluded that the low-quality surveillance data on acute encephalitis syndrome (AES)/Japanese encephalitis (JE) in Kushinagar District, India, provide little evidence to support development of prevention and control measures and to estimate the effect of interventions (*1*). Analysis of the surveillance data, however, does provide evidence supporting the effect of an ongoing intervention (i.e., JE vaccination).

In accordance with the surveillance protocol, cerebrospinal fluid and/or serum samples from AES patients admitted to the Baba Raghav Das Medical College (Gorakhpur, India) are tested for IgM against JE at the field laboratory of National Institute of Virology (NIV) at Gorakhpur (*2*). The samples are tested by using the ELISA developed by NIV Pune (Pune, India), which has a specificity of 85% (range 77%–95%) and sensitivity of 71% (range 71%–75%) (*3*). Patients with samples negative for JE are considered to have JE-negative AES.

We obtained the line-list of AES patients from the NIV laboratory at Gorakhpur for 2008–2012. Analysis of the surveillance data indicated that 251 (8.2%, range 4%–14.7%) of the 3,047 AES patients from Kushinagar were positive for JE IgM and therefore considered to be JE case-patients. JE incidence per 100,000 persons in the district declined from 2.3 cases in 2010 to 0.81 in 2011 to 0.58 in 2012 (Figure). The decline in JE incidence since 2010 could be a consequence of JE vaccination activities in Kushinagar. In 2010, a mass vaccination campaign with 1 dose of JE vaccine (SA 14–14–2 strain) was conducted among children 1–15 years of age. Subsequently, the vaccine was introduced into the childhood vaccination program as a 1-dose strategy in 2011 and a 2-dose strategy in 2013. Unfortunately, information about evaluated coverage of JE vaccine is not available from the district. On the other hand, the average annual incidence of JE-negative AES during the same period was 16 cases per 100,000 persons (95% CI 14.8–17.2), and this incidence has remained relatively stable since 2008. 

**Figure:**
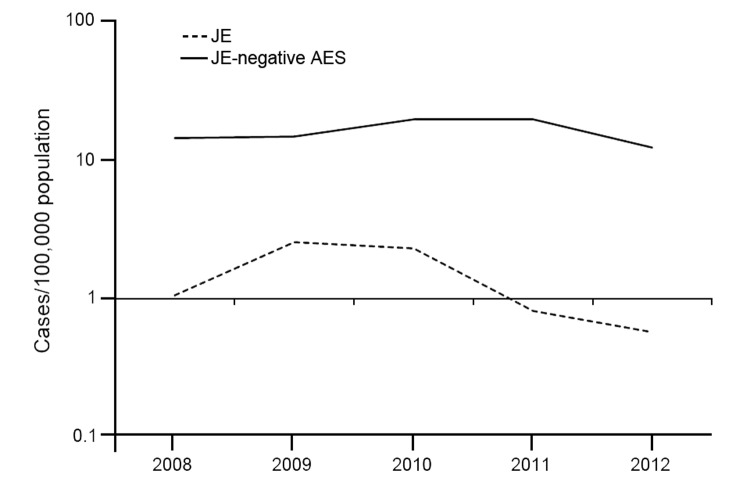
Annual incidence of Japanese encephalitis (JE) and JE-negative acute encephalitis syndrome (AES), Kushinagar District, Uttar Pradesh, India, 2008–2012.

With the isolation of enteroviruses from JE-negative AES patients, waterborne transmission has been hypothesized, and the focus of intervention has shifted toward improving sanitation and water quality. However, enteroviruses were detected only in a small proportion of AES patients. Although the quality of AES surveillance needs to be improved, as Kakkar et al. suggested (*1*), further studies are needed to understand the etiology of JE-negative AES in the district and the risk factors for transmission. These studies might include systematically investigating patients and environmental samples for enteroviral and other etiologic agents.
